# Pruritus as a Distinctive Feature of Type 2 Inflammation

**DOI:** 10.3390/vaccines9030303

**Published:** 2021-03-23

**Authors:** Simone Garcovich, Martina Maurelli, Paolo Gisondi, Ketty Peris, Gil Yosipovitch, Giampiero Girolomoni

**Affiliations:** 1 Dermatology, Università Cattolica del Sacro Cuore, 00168 Rome, Italy; ketty.peris@unicatt.it; 2 Dermatology Unit, Fondazione Policlinico Universitario A. Gemelli IRCCS, 00168 Rome, Italy; 3 Section of Dermatology and Venereology, Department of Medicine, University of Verona, 37126 Verona, Italy; maurelli.martina@gmail.com (M.M.); paolo.gisondi@univr.it (P.G.); giampiero.girolomoni@univr.it (G.G.); 4 Miami Itch Center, Dr. Phillip Frost Department of Dermatology and Cutaneous Surgery, Miller School of Medicine, University of Miami, Miami, FL 33136, USA; gyosipovitch@med.miami.edu

**Keywords:** chronic pruritus, itch, skin diseases, pruritogenic mediator, T-helper type 2 cells, interleukin 31, interleukin-4, interleukin-13, dupilumab, atopic dermatitis, prurigo

## Abstract

Pruritus is a common symptom of several skin diseases, both inflammatory and neoplastic. Pruritus might have a tremendous impact on patients’ quality of life and strongly interfere with sleep, social, and work activities. We review the role of type-2 inflammation and immunity in the pathogenesis of chronic pruritic conditions of the skin. Type 2 cytokines, including IL-4, IL-13, thymic stromal lymphopoietin, periostin, IL-31, IL-25, and IL-33 are released by mast cells, innate lymphoid cells 2, keratinocytes, and type 2 T lymphocytes, and are master regulators of chronic itch. These cytokines might act as direct pruritogen on primary sensory neurons (pruriceptors) or alter the sensitivity to other itch mediators Type 2 inflammation- and immunity-dominated skin diseases, including atopic dermatitis, prurigo nodularis, bullous pemphigoid, scabies, parasitic diseases, urticaria, and Sézary syndrome are indeed conditions associated with most severe pruritus. In contrast, in other skin diseases, such as scleroderma, lupus erythematosus, hidradenitis suppurativa, and acne, type 2 inflammation is less represented, and pruritus is milder or variable. Th2 inflammation and immunity evolved to protect against parasites, and thus, the scratching response evoked by pruritus might have developed to alert about the presence and to remove parasites from the skin surface.

## 1. Introduction

Pruritus is defined as an unpleasant sensation that provokes the desire to scratch. Pruritus is a common symptom of numerous inflammatory skin diseases, and it can be severe enough to interfere with sleeping and daily activities, and impact markedly on patients’ quality of life. Chronic pruritus (CP) is defined clinically by its persistence for more than 6 weeks and it is a frequent complaint in the general population, with a 13.5–16.8% point prevalence [1,2]. CP can be classified on the basis of its underlying etiology as dermatological, systemic, neurological, somatoform, or multifactorial [3]. The neurophysiology and basic mechanisms underlying CP were recently elucidated using animal models and with clinical studies, contributing to new pathophysiological and therapeutic concepts. CP is a common symptom of a heterogeneous spectrum of cutaneous inflammatory skin diseases, which were recently re-classified according to their immune response pattern, based on specific cellular and cytokine signatures [4].

The skin, like other epithelial barrier organs, is endowed with a complex immune cellular network, which interacts with epithelial and mesenchymal cells, to ensure and maintain barrier and homeostatic functions. The skin immunological barrier comprises a variety of resident immune cells, such as dendritic cells, macrophages, T cells, and innate lymphoid cells (ILCs) [5]. Skin resident ILCs shape the initial, innate immune response by producing effector cytokines, in response to microbial and environmental stimuli. Type-1 and type-2 immune responses are characterized by distinct cellular and cytokine repertoires, modulating skin inflammation and thus the pathogenesis of common skin disorders. Type-1 immune responses are driven by interferon (IFN)-γ to mediate cellular/cytotoxic immunity against intracellular pathogens. Type-2 immune responses are typically triggered by skin barrier disruption and have evolved to mediate host-defense against extracellular pathogens (for example, helminths and parasites) and to support tissue-repair and remodeling. In the skin, group 2 ILC (ILC2) are the main actors of type-2 immunity, producing large amounts of Th2-cytokines in response to environmental stimuli [6].

Here, we discuss the prominent role of type 2 immune responses and Th2 cytokines in the pathophysiology of chronic pruritic skin diseases. CP of dermatological origin and ‘atopic’ CP were mostly studied in the context of atopic dermatitis (AD) and chronic prurigo nodularis (PN), two itchy skin disorders with a well characterized type 2 immunopathology. Emerging clinical and experimental evidence points to an involvement of type 2 immune responses and cytokines also in other chronic itch diseases. Indeed, the skin disorders most frequently associated with CP are those characterized by type 2 inflammation or immune response, and are reviewed here, underlining the therapeutic implications.

## 2. Neurophysiology of Pruritus

The itch sensation usually originates around the dermo-epidermal junction by the activation of selective or specific nerve endings called pruriceptors. Then, itch is processed and transmitted by specific pathways, through nerves and the spinal cord, to the brain. At least two classes of primary afferent C fibers transmit itch, mechano-insensitive C fibers and mechano-responsive polymodal C-nociceptor units, as well as thinly myelinated Aδ fibers afferents [7]. Specific receptors located on these fibers can be activated by a large number of endogenous itch-inducing agents released by keratinocytes, immune cells, or neighboring neuronal afferent and exogenous agents, such as proteases released by *Staphylococcus aureus*. These sensory neurons have cell bodies in the dorsal root ganglion (DRG) and project primary afferents to the skin, and they send projections to the dorsal horn of the spinal cord, where they synapse with second- or third-order neurons that come together to form part of the spinothalamic tract, which then ascends up to the thalamus and proceed to the somatosensory and the anterior cingulate cortex [8]. Second- and third-order neurons in the spinal cord might have either excitatory or inhibitory functions [9]. Pathways of itch are complex and not fully established. Moreover, pruritus and pain pathways are largely overlapped and interrelated, though the exact relationships are still a matter of debate [10]. Several chemical pruritogens and some physical stimuli were experimentally used to elicit itch to investigate pruritus pathways and test potential anti-pruritic drugs. However, no objective methods to elicit and to evaluate itch were standardized [11].

## 3. Th2 immunity as a Driver of Chronic Pruritus

In the skin, keratinocytes (the epithelial barrier), immune cells, and sensory neurons (the pruriceptors), are in close physical proximity in an anatomical and functional niche [12,13]. Recent evidence points to a neuro-immune-epithelial cross-talk being central in both the induction and amplification of CP. This concept, the neuro-immune-epithelial interactome, was prominently investigated in the setting of atopic CP, but could be readily adapted to other Th2-driven inflammatory conditions [14]. Pruritic stimuli (exogenous or endogenous) and perturbation of skin barrier integrity cause an epithelial stress response, with keratinocytes releasing various danger-signals or alarmins [15]. These include both epithelial-derived cytokines, such as IL-1α, thymic stromal lymphopoietin (TSLP), IL-25, IL-33, proteases (kallikreins, cathepsins), and extracellular matrix (ECM) proteins, such as periostin (summarized in Table 1). Keratinocyte-derived alarmins can directly or indirectly activate cutaneous pruriceptors, thus initiating itch sensation. Interestingly, both TSLP and IL-33 were shown to directly stimulate transient receptor potential ankyrin 1 (TRPA1)+ sensory neurons, expressing the related receptors TSLPR and ST2 [16,17]. Kallikreins (KLK) of the serine protease-family promote atopic CP, by inducing skin barrier dysfunction (KLK5), activating protease activate receptor (PAR)-2 receptors on keratinocytes or sensory neurons or via non-inflammatory mechanisms (KLK7) [18,19]. Cathepsin S, another epidermal cystein protease, is involved in both AD-related inflammation and in histamine-independent itch, by activating Mas-related G-protein-coupled receptors (Mrgprs) and PAR-2 on sensory neurons [20,21]. Furthermore, the multifunctional ECM protein periostin can directly activate primary sensory afferents, via its aVβ3 integrin receptor, in the skin, inducing itch in-vivo, as a pruritogen [22]. Periostin release from keratinocytes and fibroblasts is further stimulated by TSLP and other canonical Th2-cytokines (IL-4, IL-13) in a paracrine fashion, in a vicious cycle feedback loop, as discussed in the next section [23]. Increasing evidence points to TSLP as a master switch of the epithelial–immune–neural interactome, by (a) activating pruriceptors and (b) stimulating type 2 immune cells. TSLP and the epithelial-derived alarmins IL-25 and IL-33 all promote activation of type 2 immune cells, including Th2 cells, ILC2s, Th2-cells, mast cells, basophils, and the production of Th2 cytokines, promoting inflammation, CP, and tissue remodeling.

Type 2 immune cells are strong producers of the Th2 cytokines IL-4, IL-13, and IL-31, which are expressed in AD skin and in other Th2-dominated inflammatory and pruritic skin disorders (Table 2). Indeed, IL-4, IL-13, and IL-31 are not only pro-inflammatory mediators but are strong pruritogenic cytokines [26]. In the skin, the ‘pruritogenic’ Th2 cytokines IL-4, IL-13, and IL-31 are able to directly activate selected populations of DRG sensory neurons, expressing the specific IL4-R and IL-31RA/oncostatin M receptor β subunit (OSMRβ) receptor systems [27]. In fact, pruritogenic cytokines variably induce itch in in-vivo models or humans, i.e., upon dermal injection, as in the case of IL-31 and IL-4/13 [10,11,24,32].

## 4. Th2 Cytokines, Chronic Itch Sensitization, and the Itch-Scratch Cycle

**Figure 1:**
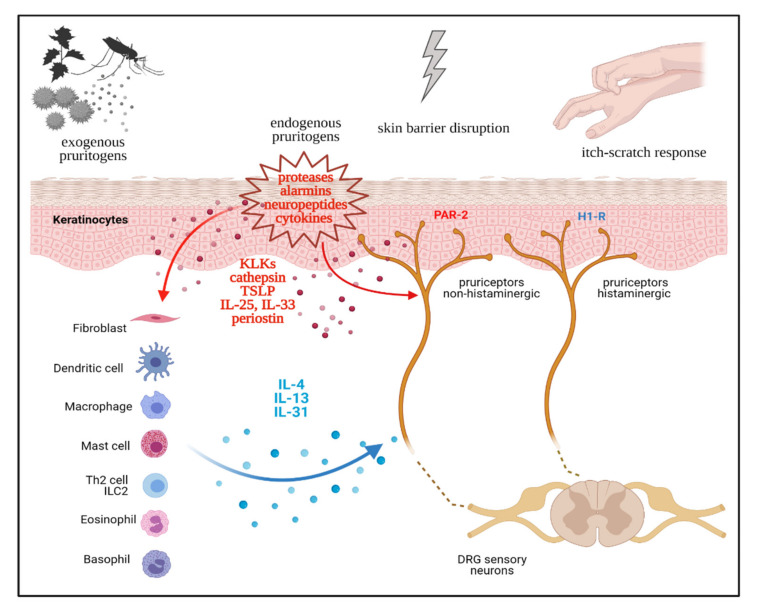
Neuro–immune–epithelial crosstalk between keratinocytes, type-2 immune cells, fibroblasts, and pruriceptors (primary sensory neurons) in the skin, mediating induction and amplification of pruritus. Created with BioRender.com.

Sensitization is a process common to both pain and itch perceptions, where enhanced neural response is triggered by lower-threshold stimuli and abnormally persists after stimulus removal [33]. The phenomenon of sensitization is a hallmark of chronic pain states and is further classified in peripheral and central aspects [34]. Similarly, sensitization to itch is increasingly studied, especially in the setting of atopic CP, with experimentally-induced itch in animal models and humans [7,35]. In analogy to pain, chronic itch sensitization might involve peripheral or central mechanisms. Clinical correlates of itch-sensitization include hyperknesis, an increased itch response to a normal pruritic stimulus, and alloknesis, an abnormal itch after application of non-pruritic (tactile) stimuli. Peripheral sensitization can be explained with a reduced activation threshold of cutaneous pruriceptors, or increased nerve fibers responsiveness and subsequent release of neurotransmitters [36]. In CP and AD patients, sensitization mechanisms seem to primarily involve lesional skin, rather than peri-lesional skin, with increased responsiveness to chemical pruritogens (histamine), mechanical, and thermal stimuli [37]. Several cellular and molecular mechanisms are involved in peripheral itch sensitization in atopic CP, including increased epidermal innervation by sensory afferents, functional adaptation of pruriceptors, and positive feedback loop mechanism in the epithelial–neuro–immune interactome [38]. Experimental and clinical evidence supports a prominent role of Th2 cytokines driving these mechanisms. Feld et al. showed than IL-31 is able to induce nerve fiber elongation and sprouting in vitro and in mice [39]. The IL-31 receptor complex (IL-31RA/OSMR) is expressed by transient receptor potential vanilloid type 1 (TRPV1)+/TRPA1+ sensory neurons of the DRG, keratinocytes, and by cutaneous immune cells (T cells, monocytes, macrophages, dendritic cells, mast cells, eosinophils, basophils) [27]. Increased epidermal innervation and aberrant activation of epidermal nerve fibers is typical of AD skin, further aggravated by barrier disruption and inflammation [40]. The IL-4 and IL-13 receptor systems are also expressed on itch-specific subsets of DRG sensory neurons, and can modulate neuronal responsiveness [41]. By employing sensory neuron-specific genetic deletion of IL-4RA, it was shown that CP is dependent on neuronal IL-4RA and JAK1 signaling [41]. Rather than triggering acute itch, activation of neuronal IL-4RA sensitizes sensory neurons to multiple other pruritogens. IL-4 significantly amplifies scratching behavior to low doses of known pruritogens like histamine [41]. In the context of atopic inflammation, IL-13 also stimulates the expression of TRPA1 channels in dermal sensory neurons and mast-cells, further supporting the importance of neuro-immune crosstalk in the skin [42]. Periostin induces itch behavior in mice, dogs, and nonhuman primates by directly activating sensory neurons through the integrin aVb3 receptor. Interestingly, TSLP induces the secretion of periostin in keratinocytes via the JAK/STAT pathway, suggesting that a TSLP-periostin mutual activation loop might be involved in chronic allergic itch [22]. In sum, type-2 immune responses and key-cytokines (IL-2, IL-4, IL-13, IL-31) are critically involved in the chronic phase of pruritus pathophysiology, mediating peripheral sensitization mechanisms and abnormal neuro–immune–epithelial cross-talk in several pruritic conditions (as depicted in Figure 1).

## 5. The Top Itchy Skin Diseases

Pruritus is associated with a heterogenous spectrum of skin diseases, including both communicable and non-communicable skin conditions. Skin diseases are increasingly re-classified with an “-omics” approach, combining both clinical–morphological and immune–pathogenetic information, to improve the traditional disease nosology and translating in molecular-targeted treatments [4,43]. Eyerich and Eyerich originally proposed an integrated, clinical and immunological classification of inflammatory skin conditions, describing four main immune response patterns. These include Th1/ILC1, Th2/ILC2, Th17/ILC3, and iTreg immune response patterns, each with distinct lymphocytic and cytokine subsets and mediating chronic inflammatory pruritic skin condition, among others. For example, the Th2/ILC2 or type 2 response is associated with “eczematous” skin conditions (such as AD, urticarial, etc.) and type 2 effector cytokines (IL-4, IL-5, IL-13, and IL-31) [44]. The most common itchy skin conditions are summarized in Table 2, on the basis of the clinical burden of pruritus and the underlying, selected immune-signatures. The mostly itchy skin disorders are driven by type 2 inflammation (Table 2). In contrast, in other Th1/ILC1, Th17/ILC3-, and iTreg-mediated skin diseases (e.g., lupus erythematosus, hidradenitis suppurativa, acne, scleroderma), pruritus is milder and inconstant. Irrespective of the original cause and underlying pathophysiology, chronic pruritic conditions determine profound suffering of the affected individuals on multiple dimensions, with significant emotional–psychological burden, sleep interference, work impairment, and loss of productivity [45]. In the next section, we describe the role of type-2 inflammation as the main driver of pruritus associated with both communicable and non-communicable skin conditions, briefly pointing at therapeutic–translational aspects (summarized in Table 3) [46].

### 5.1. Scabies

Scabies is a parasitic skin infestation transmitted by the ectoparasitic mite Sarcoptes scabiei var. hominis. Humans infected by scabies present with severe itch, usually more marked during night, and with papules and burrows that might contain mites, eggs, egg cases, fecal pellets, and debris [47,48,49]. Clinically, scabies might present as classical scabies or crusted scabies, the latter being a more severe form, typical of immunodeficient or fragile patients [50]. Adult female mites reside in the burrows within the stratum granulosum of the epidermis, inducing an inflammatory response involving mast cells, eosinophils, neutrophils, and T lymphocytes, with an increased level of total and *Sarcoptes scabiei*-specific serum IgE and IgG4. The development of the immune response corresponds to the time frame needed to develop symptoms. Mite antigens and components directly activates both the innate immune response via Toll-like receptors (TRLs) and pruriceptors (PAR-2, TRPV1, and TRPA1) [51,52]. The initial T cell response is mixed with both Th1 and Th2 components with a marked Th2 skewing, which is much more striking in crusted scabies, compared to ordinary scabies [49,50]. In addition, a Th17 immune response is active during scabies with a local release of IL-17. A strong proliferation of peripheral γδ+ T cells was reported in pig- and in cattle-models, especially in crusted scabies, with an augmented secretion of IL-17 [50,53]. Peripheral blood mononuclear cells isolated from crusted scabies secrete increased levels of Th2 cytokines, such as IL-4, IL-5, and IL-13, and decreased levels of the Th1 cytokine IFN-γ, which is more evident in crusted scabies. Additionally, in murine model of scabies, the immune response is Th2-dominated with high levels of IL-4 in lymph nodes, draining the lesional skin. The typical Th2 cytokines, IL-4 and IL-13, are overexpressed in scabies lesions, together with IL-31, which is mostly produced locally by CCR4+ Th2 cells, as well as CD163+ M2 macrophages [54]. In addition, TSLP and periostin are highly expressed in human scabies and are possibly involved in the induction of IL-31 production by M2 macrophages [54]. Several Th2 cytokines can contribute to pruritus during scabies, including IL-4, IL-13 TSLP, IL-31, and periostin. Pruritus in scabies is thus mostly mediated by non-histaminergic pathways, as shown in humans and pre-clinical models [55].

### 5.2. Cutaneous Helminth Infestations

Helminth parasites are a major cause of chronic infestations in tropical countries. They can be classified in *Nemathelminthes* (roundworms) and *Platyhelminthes* (flatworms). The Platyhelminthes can be distinguished in trematodes or flukes that cause schistosomiasis, and cestodes or tapeworms, which cause cysticercosis and echinococcosis, respectively [56]. The skin is involved during body invasion or as a target tissue, as in cutaneous larva migrans, which is the most frequent helminthic infestation of the skin. Pruritus is a common symptom of most helminthic infection. Pruritus might be present in the absence of skin findings before the onset of cutaneous lesions, and is a major reason for patients to seek medical advice [57]. Pruritus ani is characteristic of strongyloidasis and enterobiasis. Penetration of and permanence into the skin is associated with a marked localized immune response, as seen in larva migrans, cercarial dermatitis, Onchocerca volvulus, and schistosomiasis [58]. Persistence of the infestation in the skin or migration of helminths can cause a generalized immune response. A common feature of helminth infestations is the vigorous Th2 skewing of the immune response, with a dramatic expansion of Th2 lymphocyte subset, and with elevated levels of IgE, peripheral eosinophilia, and increase in tissue mast cells [59]. Indeed, Th2/IgE-mediated immune responses evolved to protect against epithelial assault by helminths and ectoparasites [60]. Stimulation of epithelial cells by helminth antigens results in the secretion of TSLP, IL-25, and IL-33. IL-25 might act as an alarmin and favor a Th2 response directed against helminths, enhancing the function of TSLP-activated dendritic cells in the induction of Th2 cells [61].

### 5.3. Insect Bite Reactions

Insect bite reactions are very commonly seen in clinical practice. Many insect types can be involved with distinct epidemiology, according to climate and geographical region, with indoor infestations persisting year-round. Reactions to insect bites can range from local to systemic, and even life threatening. After a mosquito bite, the commonly appearing sign and symptom is a whitish papule with a central small red dot. In some cases, alternatively, a skin reaction might appear beyond 24 h after the bite, with erythematous hard papules, swelling around the bite, small blisters, and purpuric discolorations [62,63]. However, some people develop severe reactions like blisters and diffuse erythematous indurations, accompanied by fever and systemic symptoms. Patients affected with lymphocytic leukemia frequently develop papulo-vesicular and nodular lesions, which represent exaggerated bite reactions, although these patients often do not recall having been bitten [62]. Insect bite reactions are typically very itchy, inducing a strong scratching response aimed at removing the insect from the skin. Immediate reactions are due to a direct effect of insect venom and salivary components on skin mast cells, which induces rapid release of preformed cytokines, such as IL-4, IL-6, and IL-13, histamine; serotonin, prostaglandins, and proteases [64,65,66]. Delayed reactions are typically manifestations of the host’s immune response to proteinaceous allergens and are typically Th2 polarized and associated with high levels of IgE [67].

### 5.4. Atopic Dermatitis

Atopic dermatitis (AD) is a common immune-mediated inflammatory skin disease, affecting up to 20% of children and 8% of adults [68]. AD manifests with skin dryness and eczematous lesions primarily affecting the face and skin folds, with a chronic-relapsing disease course. Itch is constant and might be severe enough to interfere with sleep and work/study ability [69]. Many patients affected by AD show a personal or a familiar history of atopic diseases, such as asthma, allergic rhinitis, and a specific IgE-reactivity [70]. AD is a multifactorial disease based on a genetic predisposition with the contribution of environmental factors, leading to skin barrier impairment and cutaneous inflammation, which is driven by excessive T cell activation and an increased production of inflammatory cytokines [71]. Genetic susceptibility encompasses the keratinocyte differentiation process, such as filaggrin, with a consequent skin barrier weakness, as well as immune dysregulation with a Th2 immune response. Excessive epidermal colonization by *S. aureus* is involved in pathogenesis, by damaging the skin barrier and inducing inflammatory responses and immune activation. Type 2 inflammation in AD is characterized by the activation of Th2 cells, T-cytotoxic 2 cells, innate lymphoid cells 2, eosinophils and mast cells, while other immune pathways such as Th22, Th17, Th9, and Th1 might contribute to maintain the pathogenic mechanism [71]. Multiple type 2 cytokines, including IL-4, IL-5, IL-9, IL-13, IL-25, IL-31, IL-33, and TSLP are overexpressed in skin and in the peripheral blood [72]. Interestingly, progression to the chronic phase of AD, which is characterized by CP and lichenification/tissue remodeling, is followed by an increased Th2-immune signature, with over-expression of pruritogenic cytokines (IL-13, TSLP) among others [73]. IL-13 is considered central to AD pathogenesis [74]. Indeed, blocking IL-13R or IL-13 itself with specific monoclonal antibodies (dupilumab, tralokinumab, lebrikizumab) is very effective in treating AD as well as in reducing pruritus [75]. Additionally, blocking IL-31R with nemolizumab is effective in treating pruritus associated with AD [76]. Several JAK inhibitors, specifically targeting the JAK1 pathway, which is mostly used by Th2 cytokines, are in an advanced phase of development or are already approved for the treatment of AD. Oral formulations include abrocitinib, upadacitinib, baricitinib, and gusacitinib, and are suitable to treat moderate-to-severe AD. Topical formulations include ruloxitinib, beprocitinib, and delgocitinib [77].

### 5.5. Prurigo Nodularis

Prurigo nodularis (PN), or chronic prurigo of nodular type, is an intensely itchy skin condition characterized by localized or generalized pruritic, excoriated lesions of different morphology (papules, nodules, plaque, and linear lesions) [78]. PN predominantly affects middle- to old-aged adults, with a female preponderance and ethnic distribution (African Americans) [79]. In PN patients, itch intensity is disproportionately high, leading to profound interference with sleep and work ability, leisure activities, and social relationships. A complex comorbidity profile and atopic predisposition are frequently reported in PN patients [80]. As one of the itchiest skin conditions, PN is characterized by the development of a pathological itch-scratch cycle and neuronal sensitization, leading to disease chronicity, independent of the initial disease-triggers. Due to chronic scratching, the lesional skin of PN is excoriated, hyperkeratotic, and thickened, with a mixed inflammatory infiltrate (lymphocytes, macrophages, eosinophils, neutrophils) and dermal remodeling on histology [78]. A dysregulated Th2- biased immune response is central to the pathogenesis of PN, including cutaneous upregulation of IL-31, IL-4, IL-17, and IL-22 [81,82]. Both pruritogenic alarmins TSLP and periostin are highly expressed in the PN skin and might fuel the itch-scratch cycle via feed-forward amplification loop mechanisms, as discussed previously [82,83]. Increased expression of neuropeptides (substance P, calcitonin gene-related peptide), neurotrophins (nerve growth factor and tropomyosin receptor kinase A receptor) and endothelin, support the notion of a dysregulated neuro–immune–epithelial cross-talk, also explaining the neuronal and epidermal hyperplasia of PN skin [84,85]. Dermal expression of the pruritogenic cytokine IL-31, its cognate cytokine oncostatin M, and the IL-31 receptor complex (IL31RA/OSMRβ) is increased in itchy skin of PN patients, indicating potential therapeutic targets [86]. In fact, targeted inhibition of IL-31RA and OSMRβ is currently under investigation for the treatment of PN. In line with a central role of Th2 cytokines, blocking the IL-4/13 or IL-31 signaling pathways, respectively, with dupilumab or nemolizumab, demonstrated short- and long-term efficacy in treating PN [87,88].

### 5.6. Chronic Urticaria

Chronic urticaria (CU) is a common skin disease characterized by the presence of very itchy wheals, lasting less than 24 h, for a period longer than six weeks. Mast cell derived histamine has a prominent role in the pathogenesis of urticaria and the itch associated with it. Th2 immune pathways, sustained by an increase of IL-4 and IL-5 inflammatory cytokines, were shown to induce mast cell activation and degranulation via IgE antibody production and binding to the Fcε receptor [89]. Indeed, both IL-31 and IL-33 were shown to be involved in the pathogenesis of CU, suggesting a crucial role of type 2 cytokines in the wheal and itch induction [90]. Peripheral blood basophils from chronic urticaria patients produce and release IL-31 in response to IgE-dependent stimulation, and are also responsive to IL-31 stimulation, which in turn promotes the release of IL-4 and IL-13 in an autocrine loop [91]. As a result, basophils infiltrate the skin in CU. IgE-dependent basophil activation are targeted by the monoclonal antibodies omalizumab and ligelizumab, which are both effective treatment of CU [92]. Targeting IL-31 directly, throughout nemolizumab, might prevent IL-31-mediated basophil recruitment, as well as IL-4 and IL-13 release from activated basophils. Phase II and phase III trials using dupilumab are also ongoing to test its efficacy and safety in patients with CU [92,93].

### 5.7. Bullous Pemphigoid

Bullous pemphigoid (BP) is an autoimmune subepidermal blistering disease characterized by autoantibodies targeting two hemidesmosomal proteins, transmembrane BP180, and intracytoplasmic plakin family protein BP230 [94,95]. BP mainly affects patients aged more than 60 years including people older than 80 years, and manifests with the development of tense, large, serum, or serum-hemorrhagic bullae, typically arising on the erythematous/eczematous/urticarial skin, in most cases symmetrically distributed in the lower extremities. The itch in BP is constant and severe, sometimes preceding the development of typical skin lesions, by weeks. BP is prominently induced and sustained by a Th2-driven autoimmune response [95,96]. BP180-reactive T cells produce Th2 cytokines (IL-4, IL-5, IL-6, IL-10, and IL-13), which induce the autoantibody production of the Th2-dependent IgG4 subtype in the active stages. Th2 cytokines activate eosinophils, which infiltrate skin in the presence of IL-5, with the consequent production of blisters in the presence of BP autoantibodies, which cause separation of the dermal–epidermal junction [95,96,97]. BP-associated pruritus is also mediated by IL-31 expressing eosinophils, which are a major source of this pruritogenic cytokine in the skin and serum of BP patients [98,99]. Personal experience and preliminary evidence indicates a good clinical response to dupilumab in BP patients [100]. Phase II and phase III trials using dupilumab, mepolizumab, or benralizumab (two anti-IL-5 monoclonal antibodies) are also underway to test its efficacy and safety in patients with BP [101,102].

### 5.8. Mycosis Fungoides and Sézary Syndrome

Cutaneous T-cell lymphoma (CTCL) are a heterogeneous group of non-Hodgkin’s lymphoma that primarily affects the skin [103]. Mycosis fungoides (MF) is the most common CTCL type, representing 50–73% of cases, with a prolonged clinical course that progresses over years through patch, plaque, and tumor stages [104]. Sézary syndrome (SS) is a leukemic variant of CTCL with a poor prognosis. SS clinically is characterized by diffuse erythroderma, lymphadenopathy, and peripheral blood involvement, and is typically refractory to multiple treatments [103]. Patients affected by extensive MF and particularly those with SS might complain about a persistent and devastating itch, causing a profound reduction in the quality of life [105]. MF and SS are Th2-type diseases, frequently accompanied by eosinophilia and high serum levels of IgE. Both skin and circulating tumor cells release increased IL-4, IL-5, IL-10, and IL-13. Additionally, IL-25 is highly expressed in CTCL and promotes the production of Th2 cytokines, in particular IL-13, and eosinophil infiltration. Moreover, TSLP activates dendritic cells to induce Th2-mediated inflammation, and periostin induces chronic inflammation by stimulating TSLP production in CTCL [104,106,107]. This polarized Th2 cytokine pattern is less prominent in the early stages of MF, when Th1-derived IFN-γ is frequently detected, and emerges more markedly with time, as the Th2-bias of malignant T cells [106,107]. Indeed, the same Th2-related cellular and soluble factors, such as eosinophils, kallikrein-5, and IL-31, drive the induction and persistence of CP in CTCL patients [108,109]. Through the secretion of Th2 cytokines, malignant T cells might suppress protective Th1 responses, impose a global Th2 bias, and promote susceptibility to infections [104]. Thus, targeting Th2 cytokines might be able to revert this Th2/Th1 imbalance/immunosuppression and improve CTCL-associated pruritus [110]. Indeed, dupilumab, a dual inhibitor of IL-4/IL-13 signaling, was successfully used to treat itch associated with SS in preliminary reports [111].

### 5.9. Chronic Pruritus of Unknown Origin and of the Elderly

Patients might present with long-standing CP in the absence of any underlying cutaneous, systemic, or psychiatric condition, even after extensive diagnostic workup. The term ‘chronic pruritus of unknown origin’ (CPUO) (previously known as chronic idiopathic pruritus or *pruritus sine materia*) was proposed to define this condition, whose diagnosis is made by exclusion [112]. CPUO might be localized or generalized, with increased prevalence (from 3.6 to 44.5%) among middle- to old-aged subjects [112,113]. Elderly patients are at an increased risk of CP due to the cumulative effects of aging on the skin barrier and neurotrophic function. Patients with CPUO frequently present clinical features of a ‘mild’ or ‘atopic-like’, type 2-inflammatory phenotype, including eosinophilia, weakly elevated IgE levels, and subclinical skin inflammation [114,115]. Aging-related immunosenescence might drive a Th2-immune imbalance, further aggravating the itch sensation in elderly patients. Increased IL-31 serum levels were recently reported in patients with CPUO, consistent with the hypothesis of immunosenescence-associated Th2 polarization [116]. IL-4/IL-13 antagonisms with dupilumab shows preliminary efficacy in treating CPUO [115]. Furthermore, oral JAK-inhibitors (tofacitinib) are reportedly effective in controlling refractory pruritus and scratching behavior in a small cases-series of CPUO patients [41,117]. Preliminary clinical and experimental evidence thus support the role of Th2 cytokines and related signaling pathways in the neuro-immune mechanisms of non-inflammatory CP.

## 6. Conclusions

Th2 inflammation and immunity evolved to protect against parasites, and thus the scratching response evoked by pruritus might have developed to alert about the presence and to remove parasites from the skin surface. Pruritus is the main symptom of several skin diseases, causing a relevant burden on patients’ quality of life and psychological function. In the most-itchy skin disorders, type 2 inflammation and type 2 mediators are key players both in pruritus induction and chronic sensitization. Innovative anti-pruritic treatments might rationally target single, specific itch-mediators (anti-cytokine monoclonal antibodies) or inhibit multiple pruritogenic cytokines, by blocking common signal transduction pathways (anti-JAKs). Indeed, many new systemic and topical drugs with excellent anti-pruritic activity are being investigated or are already approved for the treatment of these chronic conditions.

## Figures and Tables

**Table 1:** Principal itch mediators and their principal cells of origin.

Mediator	Source	References
IL-4	Type 2 lymphocytes, ILC2, mast cells, eosinophils, basophils	[5,7]
IL-13	Type 2 lymphocytes, mast cells, basophils, eosinophils	[5,7,24,25]
IL-31	Type 2 lymphocytes, mast cells, macrophages, dendritic cells	[26,27,28]
TSLP	Keratinocytes	[14,15,17]
IL-25	Type 2 lymphocytes, dendritic cells, macrophages, mast cells, basophils, eosinophils, keratinocytes	[14,15,29]
IL-33	Fibroblasts, mast cells, macrophages, endothelial cells, keratinocytes	[16,30]
Periostin	Fibroblasts, keratinocytes, endothelial cells	[22,23]
Proteases: kallikreins, cathepsins	Keratinocytes, mast cells, *S. aureus*	[18,19,20,21]
Histamine	Mast cells	[31]

ILC, innate lymphoid cell; TSLP, Thymic stromal lymphopoietin.

**Table 2:** Classification of common itchy skin conditions according to the clinical burden of pruritus and the underlying immune-response patterns *.

Immune Response Pattern	Th1/ILC1	Th2/ILC2	Th17/ILC3	iTreg
Pruritus burden	↑↑	↑↑↑↑	↑	↑
Cytokines	IFN-γ; TNF-α	IL-4, IL-13, IL-31	IL-17, IL-22, TNF-α	IL-10, TGF-β
Disease	Lichen planus	Scabies	Psoriasis	Scleroderma
	Graft vs. host disease, lichenoid	Cutaneous helminth infestations	Psoriasis, pustular	Systemic sclerosis
	Contact dermatitis	Insect bite reactions	Acne	Lichen sclerosus
	Drug eruption (lichenoid)	Atopic dermatitis	Hidradenitis suppurativa	Granuloma annulare
	Toxic epidermal necrolysis	Prurigo nodularis	Neutrophilic dermatoses	Keloid
	Erythema multiforme	Chronic urticaria	Rosacea	Granuloma annulare
	Cutaneous lupus erythematosus	Bullous pemphigoid	Folliculitis decalvans	Drug reaction, granulomatous
	Dermatomyositis	Cutaneous T cell lymphoma	Drug eruption, psoriasiform	Necrobiosis lipoidica
		Chronic pruritus of unknown origin		Sarcoidosis

* Table legend: classification of skin disease and immune-response patterns adapted from the original concept of Eyerich and Eyerich [4].

**Table 3:** Selected systemic agents currently in use and under development for the treatment chronic pruritic skin conditions *.

Agent	Drug Target	Phase	Indications
Dupilumab	Anti-IL-4/13R	II-IV	AD, PN, CP
Tralokinumab	Anti-IL-13	III	AD
Lebrikizumab	Anti-IL-13	III	AD
Vixarelimab	Anti-OSMRβ	II	AD, PN, CP
Tezepelumab	Anti-TSLP	II	AD
Nemolizumab	Anti-IL-31R	III	AD, PN
BMS-981164	Anti-IL-31	I	AD
MSTT1041A	Anti-IL-33R	II	AD
Etokimab	Anti-IL-33R	II	AD
Benralizumab	Anti-IL-5R	II	CU
Mepolizumab	Anti-IL-5	I-II	CU, AD
Omalizumab	Anti-IgE	IV	CU, AD
Ligelizumab	Anti-IgE mAb	III	CU
Baricitinib	JAK1/2 inhibitor	III	AD
Upadacitinib	JAK1 inhibitor	III	AD
Abrocitinib	JAK1 inhibitor	III	AD
Tradipitant	NK1R antagonist	III	AD

* adapted from [46] Abbreviations—atopic dermatitis (AD), chronic prurigo of nodular type (PN), chronic pruritus (CP), and chronic urticaria (CU).

## Data Availability

Data sharing not applicable.

## Abbreviations

Chronic pruritus (CP); atopic dermatitis (AD); innate lymphoid cells (ILCs); interferon (IFN)-γ; chronic prurigo nodularis (PN); dorsal root ganglion (DRG); thymic stromal lymphopoietin (TSLP); extracellular matrix (ECM); transient receptor potential ankyrin 1 (TRPA1); Kallikreins (KLK); protease activate receptor (PAR); Mas-related G-protein-coupled receptors (Mrgprs); oncostatin M receptor (OSMR); transient receptor potential vanilloid type 1 (TRPV1); Janus kinase/signal transducers and activators of transcription (JAK/STAT); tumor necrosis factor alpha (TNF-α); Transforming growth factor beta (TGF-β); regulatory T cells (Treg); Cutaneous T-cell lymphoma (CTCL); Mycosis fungoides (MF); Sézary syndrome (SS); chronic pruritus of unknown origin (CPUO); thymic stromal lymphopoietin (TSLP); kallikreins (KLK); protease activate receptor (PAR); histamine H1 receptor (H1-R); and dorsal root ganglion (DRG).
